# Atomic Radiations in the Decay of Medical Radioisotopes: A Physics Perspective

**DOI:** 10.1155/2012/651475

**Published:** 2012-08-14

**Authors:** B. Q. Lee, T. Kibédi, A. E. Stuchbery, K. A. Robertson

**Affiliations:** Department of Nuclear Physics, Research School of Physics and Engineering, The Australian National University, Canberra, ACT 0200, Australia

## Abstract

Auger electrons emitted in nuclear decay offer a unique tool to treat cancer cells at the scale of a DNA molecule. Over the last forty years many aspects of this promising research goal have been explored, however it is still not in the phase of serious clinical trials. In this paper, we review the physical processes of Auger emission in nuclear decay and present a new model being developed to evaluate the energy spectrum of Auger electrons, and hence overcome the limitations of existing computations.

## 1. Introduction

Unstable atomic nuclei release excess energy through various radioactive decay processes by emitting radiation in the form of particles (neutrons, alpha, and beta particles) or electromagnetic radiation (gamma-ray photons). Most of the applications using nuclear isotopes are based on the fact that the interaction of the radiations passing through material will depend on their type (photons, neutral, or charged particles) and the transferred energy. Most radioisotopes used in clinical therapy emit *β* particles, which are ionizing radiations. The biological effect is often characterized by the so-called *linear energy transfer*, LET, expressed in units of keV/*μ*m, which is a measure of the energy deposited along the particle track. A new class of radionuclides [1], including Tb^149^,  Bi^213^, Po^211^, At^211^, Ra^223^, Ac^225^, Ac^227^, Th^226^, and U^230^, which emit *α* particles (made up of two protons and two neutrons) have been considered for therapy. The LET for most therapeutic *α* emitters ranges from 25 to 230 keV/*μ*m. On the other hand, electrons and positrons emitted in nuclear *β* decay, and in the internal conversion processes, referred to here as *β* particles, have kinetic energies ranging from tens of keV to several MeV and their LET is much lower, typically ~0.2 keV/*μ*m.

**Figure 1:**
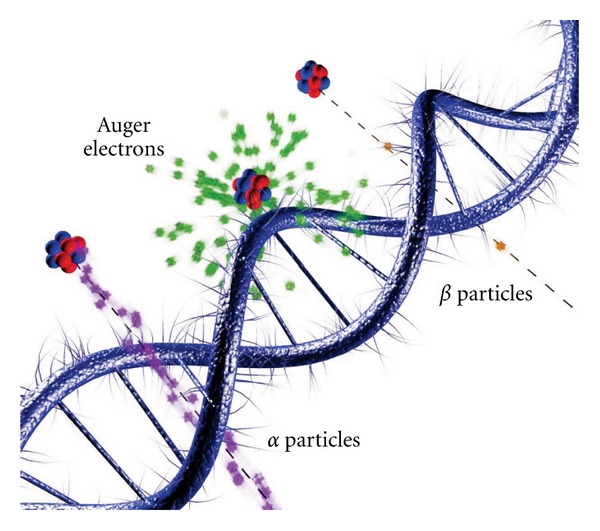
Interactions of ionizing radiations on the scale of DNA. (Courtesy of Thomas Tunningley, ANU).

A third type of ionizing radiation is Auger electrons [2], named after the French physicist Pierre Victor Auger. When an inner-shell electron is removed from an atom, the vacancy will be filled by an electron from the outer shells and the excess energy will be released as an X-ray photon, or by the emission of an Auger electron. Referred to as atomic radiations, X-ray and Auger electron emission are competing processes. The atomic transition rates, and whether X-ray or Auger emission is dominant, depend on the atomic number, the electron shells involved, and the electron configuration of the atom. The full relaxation of the inner-shell vacancy is a multistep process, resulting in a cascade of atomic radiations. The energy of emitted X-rays and/or Auger electrons depends on the atomic number, the electron shells and electron configuration involved, and is typically in the range from a few eV to 100 keV. Due to their short range (nm to *μ*m), Auger electrons with relatively low energies can have a much higher LET. For example, for electron energies below 1 keV the LET peaks at around 26 keV/*μ*m [3]. In comparison to *α* or *β* particles, Auger electrons have a much shorter range in material, which makes them ideal tools for targeted radiation therapy [4].  Figure 1 shows a pictorial comparison of the interaction sites for these three types of ionizing radiation.

Since the early 70s, when the use of Auger electrons for cancer therapy was first suggested (see the review by Howell [5]), considerable advances have been made in the understanding of the radiobiological effect of low-energy electrons. The use of Auger emitters for radiation therapy is often cited in the literature as a viable option, however the clinical research is still yet to come. According to the recent review of Buchegger et al. [4], the three main requirements of this type of targeted therapy are: (i) suitable tumor selective agent to bind the radioactive material to the tumor cells, (ii) consecutive internal irradiation cycles, and (iii) reduction of unwanted radiation damage outside the living cancer tissue. To address all these aspects would require a complex approach, however in this paper we will only focus on the physical processes required to evaluate the Auger emission in nuclear decay. We start our discussion with an overview of the current knowledge; then we propose a new approach to overcome the limitations of the current computations used for low-energy Auger emission from medical isotopes.

## 2. Radioactive Decay Processes

**Figure 2:**
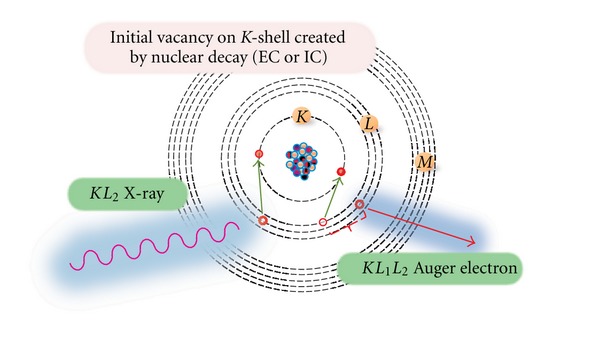
Relaxation of a vacancy in the *K* shell by X-ray and Auger emission.

When a vacancy is created in an inner electron shell, the residual atom is left in an excited state. Such a vacancy can be created by photoionization, ion-atom collisions, electron bombardment, electron capture (EC), or internal conversion electron (CE) processes. EC and CE are the only processes which involve nuclear decays and changes in nuclear structure. Typical atomic events involving the *K*-shell are shown in Figure 2.

In electron capture the nucleus decays by absorbing an atomic electron and emitting a neutrino

(1) (Z+1,A)+e→(Z,A)+νe.

The condition for electron capture decay is

(2) Eν=Q+−Ei−EX>0,

where *Q* ^+^ is the energy difference in atomic masses between parent and daughter ground states, *E*_*i*_ is the energy of the final nuclear state in the daughter nucleus, and *E*_*X*_ is the binding energy of the captured electron, *X*. The released energy, *E*_*ν*_ will be shared by the emitted neutrino and, if applicable, the Bremsstrahlung photon or shaking electron. For allowed transitions nearly all vacancies occur on the *s* shells (*K*, *L*_1_, *M*_1_, etc.) with the inner-most shells dominating. Comprehensive compilations of the relevant electron capture probability ratios for the *K*, *L*, *M*, *N*, and *O* shells were presented by Schönfeld [6]. Leaving aside the effect of *β* ^+^ decay, which may compete with electron capture, the basic relation between the subshell capture ratios (*P*_*X*_) is

(3) PK+PL+PM+PN+PO=1.

The individual terms can be calculated from their ratios. For example, for *P*_*K*_ we get

(4) PK={1+PLPK[1+PMPL(1+PNPM(1+POPN))]}−1,

and one can obtain the *P*_*L*_/*P*_*K*_ ratio as

(5) PLPK=kLK×(ΔE−EL1ΔE−EK)2,

where the *k*_*LK*_ factors are tabulated in [6], and Δ*E* is the energy difference between the parent and daughter states. For allowed and nonunique first forbidden transitions, the dominant contribution is from the *K*, *L*_1_, *M*_1_, and *N*_1_ shells, however, the contributions of the *L*_2_, *M*_2_, and *N*_2_ shells should not be neglected. The *L*_1_/*L*_2_ ratios can be calculated from amplitudes of the bound electron radial wave functions [7].

Nuclei undergoing electromagnetic decays will emit *γ*-rays, internal conversion electrons, or if the transition energy is higher than twice the electron rest mass, electron-positron pairs. In the internal conversion process an atomic electron is ejected from one of the atomic shells. The electron conversion coefficient is defined as the ratio of the probabilities of the emission of atomic electrons from shell *X* (*P*_*X*_) to the emission of *γ*-rays (*P*_*γ*_):

(6) αX=PXPγ.

The kinetic energy of the electron, *E*_CE,*X*_, can be deduced from the transition energy, *E*_tr⁡_, and the binding energy of the atomic shell, *E*_BE,*X*_, as follows:

(7) ECE,X=Etr⁡−EBE,X−Erecoil,

where *E*_recoil_ is the recoil energy of the emitting atom, which in most cases is very small. Transitions involving conversion electrons are only possible if *E*_CE,*X*_ > 0. For example, the 2.1726 keV transition from the decay of Tc^99m^ can only proceed with internal conversion from the *M*_1_ and higher shells. Theoretical internal conversion electron emission rates can be obtained from [8].

## 3. X-Rays and Auger Electrons

 It is customary to assume that the radioactive atom initially is in the neutral, ground state electronic configuration. Immediately after an electron capture or internal conversion event, the atom will be excited. In 1923, Rosseland [9] postulated that the atom relaxes via both radiative and nonradiative processes. Radiative processes will involve the emission of X-rays with characteristic energies as the atomic electrons are reorganized to fill the vacancy. In X-ray emission, an electron in an outer shell, *Y*, makes a transition to a vacancy in the inner shell, *X*, and the emitted energy of the X-ray is

(8) EXY=EBE,X−EBE,Y,

where *E*_BE,*X*_ and *E*_BE,*Y*_ are the binding energies of the atomic shells involved. The fluorescence yield, *ω*_*X*_, is defined as the number of radiative (X-ray) transitions per vacancy in any shell or subshell *X*. Considering all possible shells, subshells, *Y*, involved in filling the vacancy on the *K* shell (*X* is equal to *K*-shell), the X-ray yield, *Y*_*KY*_ can be expressed as

(9) YKY=fKωKNKY,

where *f*_*K*_ is the number of primary vacancies on the *K*-shell, and *N*_*KY*_ is the relative intensity of various X-ray transitions with ∑*N*_*KY*_ = 1.

Pierre Auger made the first confirmed experimental observation of the nonradiative process in 1925 [2]. Nonradiative processes (also called *“radiationless processes”* or the *“Auger effect”*) similarly involve the redistribution of atomic electrons but result in the emission of an atomic electron (Auger electron). The Auger electron process *XYZ* involves three electron (sub-)shells. An electron in an outer shell, *Y*, makes a transition to the vacancy in an inner shell, *X*, and an electron in outer shell *Z* is ejected. The energy of the Auger electron can be expressed as:

(10) EXYZ=EBE,X−EBE,Y−EZY,

where *E*_BE,*X*_ and *E*_BE,*Y*_ are the neutral atom binding energies for shell *X* and *Y*. *E*_*Z*_^*Y*^ is the binding energy of an electron on the *Z*-shell when the atom is already ionized with a single vacancy on the atomic shell *Y*. This process will result in vacancies in both the *Y* and *Z* shells from a single initial vacancy in the *X* shell. For example, if *X* is the *K*-shell, *Y* the *L*_1_ subshell, and *Z* the *L*_2_ subshell the electron is called a *KL*_1_ *L*_2_ Auger electron. In Coster-Kronig (CK) transitions one of the final vacancies is in the same principal shell (*Y*) as the initial vacancy (*X*). Similarly to (9) the Auger electron yield can be expressed as

(11) YKYZ=fK(1−ωK)NKYZ,

where *N*_*KYZ*_ is the relative intensity of various Auger transitions with ∑∑*N*_*KYZ*_ = 1. The sums are over all energetically possible *Y* and all possible *Z* with binding energies *E*_BE,*Y*_ ≥ *E*_BE,*Z*_.

## 4. Vacancy Propagation

The rearrangement of the atomic structure will continue until all primary, secondary, and subsequent vacancies are filled by the emission of X-rays and Auger electrons, or until no more transitions are energetically possible. In the latter case, the vacancy has reached the valence shell. This is the region where solid state, or chemical effects might be dominant. The correct treatment of such effects is beyond the scope of the present paper.

The full relaxation of the initial vacancy created in the nuclear event (Section 2) is a multistep process. While the fundamental physical picture of the individual atomic transitions remains similar to the one described above, the atomic structure will continuously change. This change will affect both the atomic-binding energies and transition rates.

Considering the number of possible atomic configurations, the procedure of evaluating the atomic radiation spectrum becomes very complex.  Table 1 compares the various calculated Auger electron yields of radioisotopes of medical importance. These include Tc^99m^, In^111^, I^123,125^, and Tl^201^. The table contains six calculations, which follow two fundamentally different approaches. The key features of the relevant physical data and assumptions are also listed and will be discussed below.

In the so-called *“deterministic approach”* (DET and DET++), the contributions from filling each vacancy are computed using closed formulae, similar to (9) and (11). Provided that all relevant transition rates are known, this approach has very small computational requirements and it was used by the Radiation Dose Assessment Resource (RADAR) [10, 11], the Decay Data Evaluation Project (DDEP) [12], and Eckerman and Endo [13]. This approach is quite reasonable and simple for transitions involving vacancies on the *K* and *L* shells. However a more realistic description must include the outer shells and hence requires that a very large number of transitions be considered. A set of approximating formulae were presented in the pioneering work of Dillman [48] to evaluate the *L-* and *M-*series atomic radiations. Dillman used a rather coarse approach, which assumed that these radiations carry a low total energy and may be treated as a *“single group”* [48]. This work led to the development of the EDISTR code [48] to evaluate the complete spectrum of atomic radiations. Recently, Endo et al. [46] have further improved the EDISTR code. In general, the accuracy of these *“deterministic predictions”* largely depends on the inclusion of outer shells.

An alternative approach is to base the calculations on *“Monte Carlo”* (MC) techniques, which prove to be better suited to the inclusion of all possible paths in the relaxation process. Such simulations begin with the selection of the nuclear decay process and the consequent creation of the initial vacancy. During the propagation of the initial vacancy, the next transition is randomly selected from all available atomic transitions, using the transition rates as weighting factors.  Table 1 includes results from Howell [14], Stepanek [15], and a very recent calculation by Pomplun [16]. As indicated in the table, the Monte Carlo approach allows the incorporation of all atomic shells with the potential to produce low-energy Auger electrons with high radiotoxicity.

Common in both approaches is the necessity to know all relevant transition energies and transition rates. All 6 calculations listed in Table 1 use transition rates from existing tabulations based on a combination of experimental data, systematics (obtained by interpolation and extrapolation), as well as theoretical calculations, which often used different assumptions, wave functions, and so forth. The two most often cited works are from Bambynek et al. [7] and the Evaluated Atomic Data Library, EADL, by Perkins et al. [30]. Most of the data presented in these compilations are for cases when there is a single vacancy on one of the atomic shells. In an effort to compensate for this limitation, the calculations presented in Table 1 have employed various corrections. One of these is the so-called Krause-Carlson correction [49], which takes into account the effect of multiple vacancies on a shell accumulated in the course of the relaxation process. Most of these calculations neglect the shakeup and shakeoff effects, which might be significant for transition rates when a vacancy is created on the outermost shells [50].

The transition energies are usually derived from atomic binding energies. As for the transition rates, the atomic binding energies are also affected by changes in the atomic configuration occurring during the relaxation process. Some of the calculations listed in Table 1 simply use neutral atom-binding energies (NAB) or semiempirical values (SE) from Larkins [40]. Others use the *Z*/*Z* + 1 rule [47] to estimate the Auger electron energies. Only the two most recent Monte Carlo approaches (Stepanek [15] and Pomplun [16]) use theoretical values obtained from relativistic Dirac-Fock calculations.

In summary, existing computations of Auger electron spectra are far from complete. Most of them are based on transition rates and transition energies obtained for single vacancies. It is also evident that the correct treatment of the relevant transition energies and rates requires a much more sophisticated computational approach than was available twenty or more years ago, when the EADL data base was developed.

## 5. New Ab Initio Calculations of Auger**** Transition Rates

The starting point to fully explore the potential of the targeted Auger-electron-based therapy is an accurate description of the relevant atomic radiation spectrum from the decaying radioisotopes. Recognizing the lack of a consistent theoretical model, the August 2011 IAEA special meeting on Intermediate-term Nuclear Data Needs for Medical Applications [51] concluded that: *“A comprehensive calculational route also needs to be developed to determine the energies and emission probabilities of the low-energy X-rays and Auger electrons to a higher degree of detail and consistency than is available at present.”* The document identifies a number of radioisotopes as potential candidates for targeted microdosimetry at the cellular level: Ga^67^, Ge^71^, Br^77^, Tc^99m^, Pd^103^, In^111^, I^123^, Nd^140^, Ta^178^, Pt^193m^, Pt^195m^, and Hg^197^. The document also concludes that for many of these isotopes further experimental studies and rigorous assessments of the existing nuclear structure information are also required.

To improve the understanding of the atomic radiation spectra in nuclear decay a new approach is required, which should use new theoretical transition energies and rates. In addressing this need we propose to adopt the following protocol for a new Monte Carlo approach.

- *Nuclear structure data* will be extracted from the Evaluated Nuclear Structure File (ENSDF) [43]. ENSDF is maintained regularly and this will ensure the use of the most up-to-date information to evaluate the nuclear event.
- *Electron capture rates* will be taken from the Schönfeld compilation [6] and subshell electron capture ratios will be calculated from (4) and (5).
- *Internal conversion coefficients* (ICC) will be taken from *BrIcc* [8]. The ICC values in that tablulation were calculated using relativistic Dirac-Fock wave functions. It is important to note that most of the previous ICC calculations assumed that the atomic vacancy created in the conversion process is filled instantly. Therefore, the conversion coefficients were calculated for the neutral atom. High-precision experimental conversion coefficients [52] indicate that the effect of the atomic vacancy should be taken into account. It is particularly important for cases when the transition energies are close to one of the shell binding energies, where the conversion coefficient is larger, and therefore the yield of atomic radiations is larger too. *BrIcc* uses the so called *“Frozen Orbital”* approximation [42] to take into account the effect of the atomic vacancy. The *BrIcc* data tables cover all atomic shells and transition energies starting from 1 keV above the shell-binding energies and continuing up to 6000 keV.
- *Auger and X-ray transition energies and rates* will be calculated using the most recent version of the General Purpose Relativistic Atomic Structure program, GRASP2K [53] and the Relativistic Atomic Transition and Ionization Properties, RATIP [54] codes. The RATIP program package was developed in the late 90s for the calculation of atomic transition and ionization properties for atoms with arbitrary charge/vacancy distributions [55], similar to those expected during the vacancy propagation process. Calculations will be carried out at every propagation step for the actual atomic configuration of the ionized atom. The calculated rates and energies together with the atomic configuration will be stored, so CPU intensive calculations need not be repeated.
- The vacancy creation and the atomic relaxation processes from *EC decay *and from *internal conversion* will be treated independently. In all practical cases IC takes place * after* the daughter atom is fully relaxed following an EC event, so internal conversion takes place in a neutral atom. This assumption is not valid in rare cases wherein the nuclear level halflife of the daughter nucleus is much shorter than the time needed for the atom to fully relax. For example, a shift of 20 ± 7 eV of the *K* conversion electron line of the 963 keV transition in the electron capture decay of Eu^152m^ [56] is one of the few experimental observations of this rare scenario. Depending on the level scheme, the radioactive decay may produce multiple electromagnetic transitions, which depending on the conversion coefficient may proceed with the emission of multiple conversion electrons. There is a finite, usually very small probability, that a second conversion electron is emitted before the vacancy created in the first conversion process could fully relax. Based on the average nuclear and atomic halflives, it is a very unlikely event, and it will not be considered in our model.
- The *ab initio treatment of the propagation process* including the random sampling of the available decay channels will ensure the realistic evaluation of the atomic spectra. A key element of the proposed model is the use of transition energies and transition rates calculated for the given atomic configuration (see (d) above). While in terms of computing requirements this is an expensive approach, it should improve the accuracy of the model.
- The *atom is assumed to be free*, and any influence from the chemical environment or solid-state effects is neglected. In our model the propagation of a particular vacancy will be terminated if there is no higher state energetically available, or if it has reached the valence shell. However the propagation of the event is not complete while there are any inner vacancies still left; the propagation of these vacancies will continue until all have reached the valence shell. In contrast, Howell [14] has assumed that once a vacancy reaches the valence shell, it will be immediately neutralized by absorbing electrons from the neighboring environment. There is strong evidence that this assumption is not correct. Specifically, the Auger cascade takes about 10^−16^ to 10^−14^ s to complete, and as pointed out recently by Robertson [57] and Pomplun [16], the proposed neutralization process is too slow to have an effect on the much faster propagation process. In the proposed model it will therefore be assumed that the vacancies on the valence shell(s) will remain unfilled throughout the entire atomic relaxation process. The atomic processes involved in neutralization of the ionized atom at the end of the above vacancy propagation would go beyond the scope of the present study.

It also should be noted that in many medical applications, the radioactive nuclide is attached to a molecule, which will affect the atomic transition energies and rates, particularly for the outermost shells. None of the previous calculations listed in Table 1 consider this effect. The relativistic multiconfiguration Dirac-Fock method we propose to use has the potential to incorporate the chemical environment, however, at least initially, this option will not be considered in our model.

## 6. Pilot Study

To explore the implications of this new approach, a *pilot model* was developed. This model follows the proposed approach, except that fixed atomic transition rates were taken from the EADL [30] data base. EADL contains X-ray, Auger electron (including Coster-Kronig and super Coster-Kronig) transition probabilities and energies for an atom with a single vacancy. These were calculated with a Dirac-Hartree-Slater method using *j-j* coupling. These calculations use semiempirical corrections to improve the accuracy for low-energy Coster-Kronig transitions. Since the EADL contains the most complete atomic data available to date, and since it describes the total transition rates correctly, it was adopted for the pilot model. However, the transition energies from EADL have been replaced with values deduced from binding energies calculated at each propagation step using the RAINE Dirac-Fock code [42]. The RAINE code tends to slightly overestimate the binding energies of the inner shells and this results in some of the K Auger lines appearing above their experimental values [57, 58]. Transitions with negative energies, that is, energetically not allowed, were excluded. This approach, at least on the superficial level, takes into account the effect of the presence of multiple vacancies and should improve the accuracy of the transition energies.

**Figure 3:**
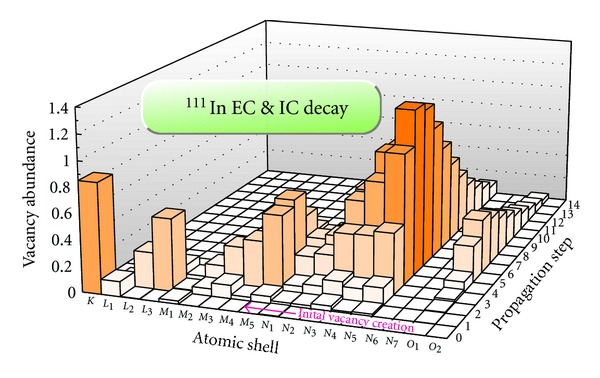
Vacancies created during the relaxation process in In^111^.

Using the pilot model, detailed calculations have been carried out for two of the isotopes listed in Table 1: Tc^99m^ and In^111^.  Figure 3 shows the abundance of atomic vacancies for each atomic shell during the atomic vacancy cascade. Vacancy creation from the nuclear decay occurs at step “0,” and events with up to 14 propagation steps are indicated. The plot was generated by evaluating 1,000,000 EC decays of In^111^, one of the commonly used radioisotopes for nuclear imaging. More than 97.5% of the initial vacancies are from electron capture on the *K*- and *L*_1_-shells. Closer examination of the graph reveals how the vacancies *“migrate”* towards the outer shells. For most of the events, the created vacancies take 7 or 8 propagation steps to reach the outer shells. Beyond that number of propagation steps, the vacancy abundance in Figure 3 shows a decrease because events with more steps become increasingly unlikely. Some key features of the propagation process include: the highest abundances of the vacancies are on the last subshell of each principal shell: *L*_3_, *M*_5_, and *N*_5_. (*N*_6_ and *N*_7_ are not occupied.) As the vacancies approach the outer shells (*M* and *N*) they are retained longer; that is, they are more likely to survive for several propagation steps.

In Tables 2 and 3, the nuclear and atomic transition energies and yields obtained for Tc^99m^ and In^111^ are compared with literature values from RADAR [10, 11], DDEP [12], Eckerman & Endo [13], Howell [14] and Stepanek [15, 59]. Our values are given in the last column of the tables, which are based on 10 million (^99m^Tc) and 1 million (^111^In) Monte Carlo events. Each entry of these tables consists of two rows. Transition energies (in keV) are given in the first row and transition probabilities (in units of emission per nuclear decay) are in the second row. The contribution of internal conversion for each atomic subshell is evaluated, however the summary tables presented here only give values averaged for the principal shells. The electron capture decay of In^111^ will also produce atomic vacancies. The electron capture events are not listed explicitly in Table 3, but the associated atomic radiations are fully accounted for in Table 3.

The main part of Tables 2 and 3 is concerned with the atomic radiations. One of the benefits of the Monte Carlo approach is the ability to consider all possible transitions, providing that they are energetically allowed and the corresponding transition rates are known. In the present computation for each transition, the type (Auger electron or X-ray), the emitted energy, and the corresponding full atomic configuration is stored on disk. A separate program is used to extract the average energy and yield for any transition type of interest. The grouping of the atomic transitions follows the convention established in previous studies. While for the transitions involving vacancies on the *K* and *L* shells there is a general agreement between our values and most of the other calculations, for the other shells it is evident that either no data is given in previous studies, or their energies and/or rates are different.

The most notable difference is for the *NXY* Auger electrons in the EC decay of In^111^ between our pilot model and Stepanek [15, 59] and Howell [14]. The later one can be attributed to the so-called *“fast neutralization”* approach resulting in significantly larger numbers of Auger electrons. By filling the valence vacancies instantly, fast neutralization creates significantly more opportunities for other vacancies to be filled by Auger processes, especially in large atoms. The slower neutralization approach in the present study, coupled with consideration of the charge distribution at each stage of the cascade, recognises that many of the *NXY* Auger and *NNX* Coster-Kronig transitions become energetically impossible once the atom has lost a few electrons. Some X-ray transitions are available to take their place. Further studies are required to explore both experimentally and theoretically the full extent of the atomic relaxation process leading to the full neutralization of the atom.

**Figure 4:**
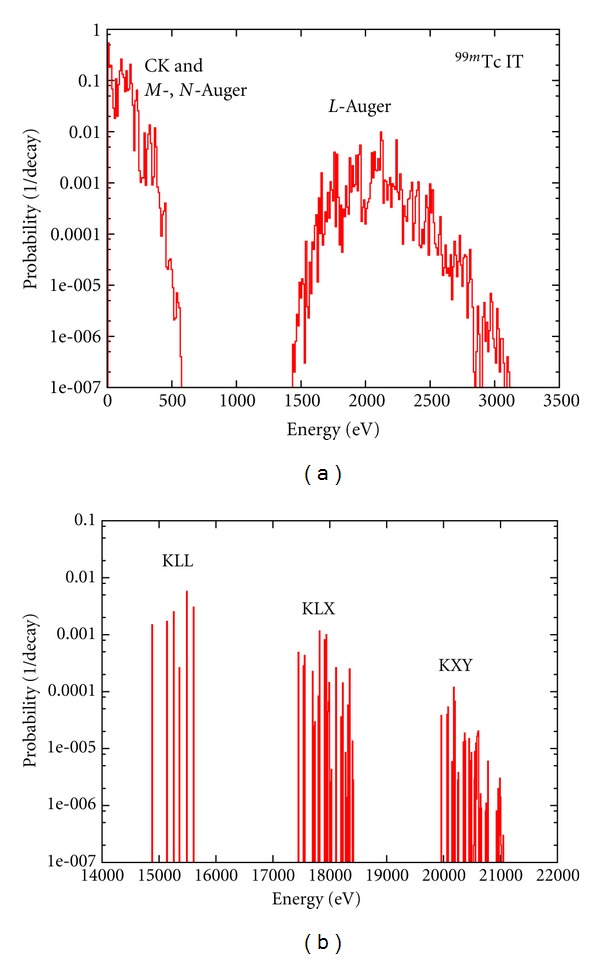
Calculated energy spectrum of Auger electrons in the decay of Tc^99m^. The vertical axis is the probability per nuclear decay for a 10 eV energy bin. (a) is the low-energy (0–3500 eV), and (b) shows the *K*-shell Auger lines.

**Figure 5:**
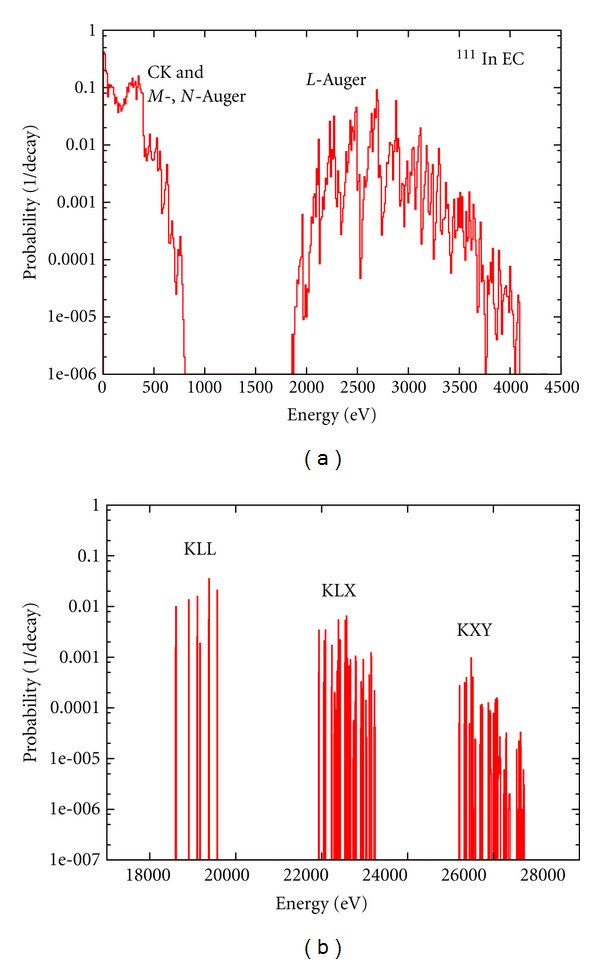
Calculated energy spectrum of Auger electrons in the decay of In^111^. The vertical axis is the probability per nuclear decay for a 10 eV energy bin. (a) is the low-energy (0–4500 eV) and (b) shows the *K*-shell Auger lines.

The calculated Auger electron energy spectra for Tc^99m^ and In^111^ are shown in Figures 4 and 5. In the case of Tc^99m^ more than 450 transition types (10) have been computed. Most transition types have multiple satellite lines at a range of energies corresponding to different atomic configurations. For the Tc^99m^ our computations resulted in a spectrum with more than 87000 Auger lines. For clarity, a 10 eV energy bin was used in these plots and the frequency of the transitions was converted to *yields per nuclear decay*. Apart from the work of Eckerman and Endo [13], energy spectra have never been calculated for radioisotopes listed in Table 1. Only a few experimental investigations exist on the detailed energy spectrum of these Auger electron emitters. The only known Auger-electron spectra measured for Tc^99m^ [60] and In^111^ [61] cover a relatively high-energy range: 1.50–2.32 keV (^99m^Tc) and 1–6 and 15–35 keV (^111^In). While for some energy regions (1.5–4 keV for In^111^) the experimental spectra are in agreement with our calculations, detailed experimental spectra are required to benchmark our calculations. This is particularly important for low Auger energies (*E* < 1 keV), which have the largest potential for targeted Auger therapy [4].

An important result of the pilot model is the calculated total yield of Auger electrons: Tc^99m^: 3.37 and In^111^: 5.75 electrons per radioactive decay of the parent atom. In accord with our assumption that valence-shell vacancies persist, these results are consistent with those of Pomplun [16]. We have therefore demonstrated that our calculations using the *pilot model* can reproduce the previous Monte Carlo calculations for these isotopes.

## 7. Conclusions

 There is continuing interest in medical applications of Auger electrons which accompany nuclear decay, particularly for the targeted treatment of cancer cells at the DNA scale. In most cases these applications are based on theoretical predictions of the emitted Auger and X-ray spectra. As it is evident from Table 1, there is a significant difference in the Auger yields reported in the literature over the last 20 years. Most of this difference can be attributed to the lack of detailed knowledge of the relevant atomic transition rates, most prominently in the outer (*M*, *N*, etc.) shells. Simplistic assumptions regarding the atomic configurations during the intermediate steps of vacancy propagation and the incomplete treatment of the effect of multiple vacancies also limit the validity of earlier calculations.

We are developing a new model using *ab initio* calculations based on the relativistic Dirac-Fock approach and Monte Carlo techniques, which has the potential to overcome these limitations. Pilot calculations for the isotopes Tc^99m^ and In^111^, based on fixed transition rates from the EADL database [30], are in satisfactory agreement with previous computations.

## Figures and Tables

**Table 1:** Calculated Auger electron yields for selected medical radioisotopes.

	RADAR [10, 11]	DDEP [12]	Eckerman and Endo [13]	Howell [14]	Stepanek [15]	Pomplun [16]	Present study
Nuclear decay data^(a)^	ENSDF	DDEP	ENSDF	ENSDF	ENSDF	ICRP38	ENSDF
Conversion coefficients	[17]	[8, 18]	[18, 19]	[18]	[15]	[17, 20, 21]	[8]
Electron capture ratios	[22]	[23]	[24]	[22, 25]	[22, 25]	[22]	[23]
Atomic shells	K, L	K, L	K–O	K–O	K–N	K–N	K–R
Atomic transition rates^(b)^	[7, 26]	[27–29]	[30, 31]	[32–35] (A)	[30]	[32–34] (A)	[30]
	RADLST	EMISSION	EDISTR04	[27, 36] (X)		[37, 38] (X)	
Atomic transition energies^(c)^	NAB [39]	SE [40]	NAB [30]	*Z*/*Z + *1(A)	DF	DF [41]	DF [42]
				NAB (X)			
Vacancy propagation^(d)^	DET	DET	DET++	MC	MC	MC	MC
Charge neutralization	No	No	No	Yes	No	No	No

	Auger electron yield per nuclear decay						

^99m^Tc (6.007 h)	0.122	0.13	4.363	4.0		2.5	3.37
^ 111^In (2.805 d)	1.136	1.16	7.215	14.7	6.05		5.75
^ 123^I (13.22 h)	1.064	1.08	13.71	14.9		6.4	
^ 125^I (59.4 d)	1.77	1.78	23.0	24.9	15.3		
^ 201^Tl (3.04 d)	0.773	0.614	20.9	36.9			

^(a)^ENSDF: evaluated nuclear structure file [43]; DDEP: decay data evaluation project [12]; ICRP38: international commission on radiological protection [44].

^(b)^Computer codes: RADLST by Burrows [26], EMISSION by Schönfeld and Janßen [45], and EDISTR04 by Endo et al. [46]; (A): Auger electrons, (X): X-rays.

^(c)^Transition energies deduced from: NAB: neutral atom binding energies; SE: semiempirical Auger energies *Z/Z +* 1 approximated from neutral atom binding energies [47]; DF: relativistic Dirac-Fock calculations.

^(d)^Approach to treat vacancy cascades: DET: deterministic, using closed formulae; DET++: deterministic, using up to 3000 possible transitions; MC: Monte Carlo approach.

**Table 2:** Average radiation yields and energies of ^99m^Tc. For every entry the first line contains the energies in keV, and the second line (in italics) contains the emission probabilities.

	RADAR [10, 11]	DDEP [12]	Eckerman and Endo [13]	Howell [14]	Pomplun [16]	Present study (pilot model)
Nuclear radiations						
*γ*_1_		2.1726(4)			2.1	2.1726(4)
		*7.4(2)E* − 9			*1.05E* − 2	*7.3(2)E* − 9
CE − M	1.6	[1.628 : 1.919]	1.748	1.82	1.779 ^(a)^	1.781
	*7.46E − 1*	*8.80(24)E − 1 *	*8.62E − 1 *	*9.91E − 1 *	*9.14E − 1 *	*8.75E − 1*
CE − N		[2.104 : 2.170]	2.173		2.060 ^(a)^	2.139
		*1.17(3)E − 1*	*1.30E − 1 *		*7.53E − 2 *	*1.15E − 1 *
CE − O						2.166
						*2.50E − 6 *
*γ*_2_	140.5	140.511(1)	140.5	141	140.5	140.511(1)
	*8.906E − 1*	*8.85(2)E − 1 *	*8.91E − 1 *	*8.89E − 1 *	*9.012E − 1 *	*8.906E − 1 *
CE − K	119.5	119.467(1)	119.5	119	119.4	119.467
	*8.80E − 2*	*9.20(27)E − 2 *	*8.92E − 2 *	*8.43E − 2 *	*8.440E − 2 *	*8.79E − 2 *
CE − L	137.5	[137.468 : 137.834]	137.5^(a)^	137	137.4^(a)^	137.494
	*1.07E − 2*	*1.142(35)E − 2 *	*1.087E − 2 *	*1.36E − 2 *	*1.14E − 2 *	*1.07E − 2 *
CE − M	140.0	[139.967 : 140.258]	140.1	140	140.1^(a)^	139.977
	*1.9E − 3*	*2.09(6)E − 3 *	*1.99E − 3 *	*3.70E − 3* ^(b)^	*2.70E − 3 *	*1.94E − 3*
CE − N				140.5	140.4	140.4
				*3.80E − 4 *	*3.00E − 4 *	*3.13E − 4 *
CE − O						140.5
						*2.13E − 5 *
*γ*_3_	142.6	142.683(1)				142.683(1)
	*2E − 4*	*2.3(2)E − 4 *				*2.5(2)E − 4 *
CE − K	121.6	121.631(25)	121.6	122		121.586
	*5.5E − 3*	*6.7(6)E − 3 *	*5.50E − 3 *	*5.90E − 3 *		*6.51E − 3 *
CE − L	139.6	[139.632 : 139.998]	139.8^(a)^	140		139.741
	*1.7E − 3*	*2.15(20)E − 3 *	*1.75E − 3 *	*2.50E − 3 *		*2.05E − 3 *
CE − M	142.1		142.2			142.140
	*3E − 4*		*3.48E − 4 *			*4.00E − 4 *
CE − N						142.57
						*6.12 E − 5*
CE − O						*142.62 *
						*1.50E − 6 *

X-rays						
K*α*_1_	18.4	18.3672	18.33	18.4	18.36	18.421
	*4.02E − 2 *	*4.21(12)E − 2 *	*4.06E − 2 *	*3.89E − 2 *	*3.65E − 2 *	*4.05E − 2 *
K*α*_2_	18.3	18.251	18.21	18.3	18.24	18.302
	*2.10E − 2 *	*2.22(7)E − 2 *	*2.14E − 2 *	*2.17E − 2 *	*1.96E − 2 *	*2.13E − 2 *
K*β*	20.6	20.677^(a)^	20.59	20.7^(a)^	20.7^(a)^	20.729
	*1.20E − 2 *	*1.30(4)E − 2 *	*6.53E − 3* ^(c)^	*1.18E − 2 *	*9.10E − 3 *	*1.18E − 2 *
L	2.4	[2.134 : 3.002]		2.45	2.499	2.466
	*4.8E − 3*	*4.82(12)E − 3*		*4.90E − 3*	*4.2E − 3*	*4.72E − 3*
M				0.236		0.263
				*1.20E − 3*		*7.83E − 4*
N						0.047
						*8.73E − 1*

Auger electrons						
KLL		[14.86 : 15.58]	15.42	15.3	15.3	15.37
		*1.49(6)E − *2	*1.48E − *2	*1.26E − *2	*1.42E − *2	*1.48E − *2
KLX		[17.43 : 18.33]	17.82	17.8	17.83	17.85
		*2.79(10)E* * − 3*	*5.59E* *−3*	*4.70E* *−3*	*4.60E* *−3*	*5.58E* *−3*
KXY		[19.93 : 21.00]			20.32	20.27
		*2.8(1)E − * *4*			*4.0E − * *4*	*5.07E − * *4*
K total	15.5					16.15^(a)^
	*2.07E − * *2*	*2.15(8)E − * *2 *	*2.04E − * *2 *	*1.73E − * *2 *	*1.92E − * *2 *	*2.08E − * *2 *
CK LLM						0.054
						*9.20E − * *3*
CK LLX				0.0429	0.1721	0.144
				*1.93E − * *2*	*1.13E − * *2 *	*9.48E − * *3 *
LMM			2.054	2.05	2.032	2.016
			*9.03E − * *2 *	*8.68E − * *2 *	*8.47E − * *2 *	*9.02E − * *2 *
LMX			2.333	2.32	2.326	2.328
			*1.41E − * *2 *	*1.37E − * *2 *	*1.10E − * *2 *	*1.41E − * *2 *
LXY				2.66	2.631	2.654
				*1.20E − * *3 *	*6.00E − * *4 *	*6.07E − * *4 *
L total	2.2	[1.6 : 2.9]	2.09^(a)^	1.77^(a)^	1.86^(a)^	1.765
	*1.02E − * *1* ^(d)^	*1.089(9)E − * *1* ^(d)^	*1.04E − * *1 *	*1.21E − * *1 *	*1.08E − * *1 *	*1.24E − * *1 *
CK MMX			0.1142	0.116	0.09578	0.104
			*7.09E − * *1*	*7.47E − * *1*	*3.49E − * *1*	*7.10E − * *1*
MXY			0.2061	0.226	0.1818	0.170
			*1.08E + * *0 *	*1.10E + * *0 *	*1.116E + * *0 *	*1.10E + * *0 *
Super CK NNN						0.014
						*5.36E − * *1*
CK NNX			*0.02961 *	*0.0334 *	*0.01291 *	*0.012 *
			*2.47E + * *0 *	*1.98E + * *0 *	*8.723E − * *1 *	*8.45E − * *1 *

Total energy release per nuclear decay (keV)						
*γ*-rays				124.997		125.133
CE electrons				15.383		15.232
X-rays				1.367		1.433
Auger electrons				0.899		0.833

^(a)^Evaluated from subshell data.

^(b)^M-, N-shell summed contribution.

^(c)^K*β*1 only.

^(d)^Auger electrons only, does not include Coster-Kronig transitions.

**Table 3:** Average radiation yields and energies of ^111^In. For every entry the first line contains the energies in keV, and the second line (in italic) contains the emission probabilities.

	RADAR [10, 11]	DDEP [12]	Eckerman and Endo [13]	Howell [14]	Stepanek [15, 59]	Present study (pilot model)
Nuclear radiations						
*γ*_1_		150.81(3)				150.81(3)
		*1.5(15)E − 5*				*~3E − 5*
*γ*_2_	171.3	171.28(3)	171.3	171		171.28(3)
	*9.02E − 1*	*9.061(20)E − 1 *	*9.06E − 1 *	*9.06E − 1 *		*9.065(25)E − 1 *
CE − K	144.6	144.57(3)	144.6	145		144.57
	*7.80E − 2*	*8.13(20)E − 2 *	*8.51E − 2 *	*8.24E − 2 *		*8.39E − 2*
CE − L	167.3	[167.3 : 167.7]	167.3^(a)^	167		167.29
	*1.06E − 2*	*1.02(3)E − 2 *	*1.08E − 2 *	*1.00E − 2 *		*1.05E − 2 *
CE − M	170.5	[170.51 : 170.88]	170.7	171		170.52
	*2.0E − 3*	*1.97(5)E − 3 *	*2.09E − 3 *	*1.40E − 3* ^(b)^		*2.01E − 3 *
CE − N+	171.2		171.3			171.18
	*4E − 4*		*4.35E − 4 *			*3.92E − 4 *
CE − N						171.18
						*3.70E − 4*
CE − O						171.27
						*2.40E − 5 *
*γ*_3_	245.4	245.35(4)	245.4	245		245.35(4)
	*9.40E − 1*	*9.412(6)E − 1 *	*9.41E − 1 *	*9.37E − 1 *		*9.409(18)E − 1 *
CE − K	218.7	218.64(4)	218.7	219		218.64
	*4.93E − 2 *	*4.93(10)E − 2 *	*5.04E − 2 *	*5.21E − 2 *		*5.03E − 2 *
CE − L	241.4	[241.33 : 241.81]	241.5^(a)^	241		241.46
	*7.9E − 3 *	*7.70(15)E − 3 *	*7.96E − 3 *	*9.10E − 3 *		*7.89E − 3 *
CE − M	244.6	[244.58 : 244.95]	244.7	245		244.63
	*1.5E − 3*	*1.50(3)E − 3 *	*1.56E − 3 *	*1.90E – 3* ^(b)^		*1.51E − 3 *
CE − N+	245.3		245.4			245.26
	*3E − 4*		*3.09E − 4 *			*2.70E − 4 *
CE − N						245.26
						*2.58E − 4*
CE − O						245.34
						*1.20E − 5*

X-rays						
K*α*_1_	23.2	23.1739	23.15	23.2	23.3	23.25
	*4.433E − 1 *	*4.447(26)E − 1 *	*4.50E − 1 *	*4.63E − 1 *	*4.58E − 1 *	*4.51E − 1 *
K*α*_2_	23.0	22.9843	22.96	23.0	23.1	23.06
	*2.350E − 1 *	*2.365(18)E − 1 *	*2.40E − 1 *	*2.40E − 1 *	*2.37E − 1 *	*2.39E − 1 *
K*β*	26.1	26.19^(a)^	26.25^(a)^	26.2^(a)^	26.3^(a)^	26.26
	*1.450E − 1 *	*1.466(16)E − 1 *	*7.87E − 2 *	*1.37E − 1 *	*1.48E − 1 *	*1.42E − 1 *
L	3.1	[2.77: 3.95]		3.23	3.25	3.23
	*6.90E − 2*	*6.78(14)E − 2*		*4.99E − 2*	*7.83E − 2*	*6.90E − 2*
M				0.356	0.431	0.424
				*3.00E − 3*	*2.50E − 3*	*2.54E − 1*
N+					0.0521	0.068
					*7.75E − 1*	*1.03E + 0*

Auger electrons						
KLL		[18.675: 19.636]	19.28	19.1	19.3	19.23
		*1.05(3)E − 1 *	*1.06E − 1 *	*1.03E − 1 *	*9.84E − 2 *	*1.07E − 1 *
KLX		[21.923: 23.172]	22.42	22.3	22.5	22.46
		*4.5(2)E − 2 *	*4.37E − 2 *	*3.94E − 2 *	*4.35E − 2 *	*4.39E − 2 *
KXY		[25.171: 26.028]	25.58	25.5	25.7	25.64
		*5(1)E − 3*	*4.33E − 3*	*3.60E − 3*	*4.10E − 3*	*4.29E − 3*
K total	19.3					20.3
	1.56*E* − 1					*1.55E − 1*
CK LLM						0.032
						*4.82E − 2*
CK LLX				0.183	0.247	0.234
				*1.51E − 1*	*1.52E − 2*	*1.32E − 1 *
LMM			2.611	2.59	2.60	2.58
			*8.16E − 1 *	*8.35E − 1 *	*8.03E − 1 *	*8.16E − 1 *
LMX			3.054	3.06	3.06	3.06
			*1.88E − 1 *	*1.90E − 1 *	*1.81E − 1 *	*1.88E − 1 *
LXY			3.515	3.53	3.54	3.54
			*1.14E − 2 *	*1.09E − 2 *	*1.05E − 2 *	*1.13E − 2 *
L total	2.7	[3.404: 3.804]				2.31
	*9.80E – 1* ^(c)^	*1.005(8)E + 0* ^(c)^				*1.20E + 0*
CK MMX			0.1280	0.125	0.0103	0.098
			*8.86E − 1 *	*9.15E − 1 *	*8.57E − 1 *	*8.59E − 1 *
MXY			0.3454	0.350	0.328	0.308
			*2.12E + 0 *	*2.09E + 0 *	*2.05E + 0 *	*2.12E + 0 *
Super CK NNN						0.020
						*5.38E − 1*
CK NNX			0.03677	0.0388	0.0268	0.017
			*3.04E + 0 *	*2.54E + 0 *	*1.49E + 0 *	*6.81E − 1 *
NXY				0.00847	0.0518	0.054
				*7.82E + 0 *	*3.63E − 1 *	*2.06E − 1 *

Total energy release per nuclear decay (keV)						
*γ-*rays				366.532^(d)^		386.154
CE electrons				25.957		27.657
X-rays				19.966		19.994
Auger electrons				6.750		6.678

^(a)^Evaluated from subshell data.

^(b)^M-, N-shell summed contribution.

^(c)^Auger electrons only, does not include Coster-Kronig transitions.

^(d)^Possible misprint in the original paper, should read 386.532 keV.
